# Tsetse fly tolerance to *T. brucei* infection: transcriptome analysis of trypanosome-associated changes in the tsetse fly salivary gland

**DOI:** 10.1186/s12864-016-3283-0

**Published:** 2016-11-25

**Authors:** Irina Matetovici, Guy Caljon, Jan Van Den Abbeele

**Affiliations:** 1 Unit of Veterinary Protozoology, Department of Biomedical Sciences, Institute of Tropical Medicine Antwerp (ITM), Antwerp, Belgium; 2 Present address: Laboratory of Microbiology, Parasitology and Hygiene (LMPH), Department of Biomedical Sciences, University of Antwerp, Wilrijk, Belgium

**Keywords:** Tsetse fly, Salivary gland, *Trypanosoma brucei*, RNA-seq, Tolerance

## Abstract

**Background:**

For their transmission, African trypanosomes rely on their blood feeding insect vector, the tsetse fly (*Glossina* sp.). The ingested *Trypanosoma brucei* parasites have to overcome a series of barriers in the tsetse fly alimentary tract to finally develop into the infective metacyclic forms in the salivary glands that are transmitted to a mammalian host by the tsetse bite. The parasite population in the salivary gland is dense with a significant number of trypanosomes tightly attached to the epithelial cells. Our current knowledge on the impact of the infection on the salivary gland functioning is very limited. Therefore, this study aimed to gain a deeper insight into the global gene expression changes in the salivary glands of *Glossina morsitans morsitans* in response to an infection with the *T. brucei* parasite. A detailed whole transcriptome comparison of midgut-infected tsetse with and without a mature salivary gland infection was performed to study the impact of a trypanosome infection on different aspects of the salivary gland functioning and the mechanisms that are induced in this tissue to tolerate the infection i.e. to control the negative impact of the parasite presence. Moreover, a transcriptome comparison with age-matched uninfected flies was done to see whether gene expression in the salivary glands is already affected by a trypanosome infection in the tsetse midgut.

**Results:**

By a RNA-sequencing (RNA-seq) approach we compared the whole transcriptomes of flies with a *T. brucei* salivary gland/midgut infection versus flies with only a midgut infection or versus non-infected flies, all with the same age and feeding history. More than 7500 salivary gland transcripts were detected from which a core group of 1214 differentially expressed genes (768 up- and 446 down-regulated) were shared between the two transcriptional comparisons. Gene Ontology enrichment analysis and detailed gene expression comparisons showed a diverse impact at the gene transcript level. Increased expression was observed for transcripts encoding for proteins involved in immunity (like several genes of the Imd-signaling pathway, serine proteases, serpins and thioester-containing proteins), detoxification of reactive species, cell death, cytoskeleton organization, cell junction and repair. Decreased expression was observed for transcripts encoding the major secreted proteins such as 5′-nucleotidases, adenosine deaminases and the nucleic acid binding proteins Tsals. Moreover, expression of some gene categories in the salivary glands were found to be already affected by a trypanosome midgut infection, before the parasite reaches the salivary glands.

**Conclusions:**

This study reveals that the *T. brucei* population in the tsetse salivary gland has a negative impact on its functioning and on the integrity of the gland epithelium. Our RNA-seq data suggest induction of a strong local tissue response in order to control the epithelial cell damage, the ROS intoxication of the cellular environment and the parasite infection, resulting in the fly tolerance to the infection. The modified expression of some gene categories in the tsetse salivary glands by a trypanosome infection at the midgut level indicate a putative anticipatory response in the salivary glands, before the parasite reaches this tissue.

**Electronic supplementary material:**

The online version of this article (doi:10.1186/s12864-016-3283-0) contains supplementary material, which is available to authorized users.

## Background

A group of devastating vector-borne parasitic diseases, African trypanosomiasis in sub-Saharan Africa, is caused by protozoan parasites of the genus *Trypanosoma*, including two human-pathogenic species of the *T. brucei* complex. The key of the transmission of these parasites is their specific biological relationship with an exclusive blood feeding insect, the tsetse fly (*Glossina* spp.). Indeed, tsetse fly is an obligatory intermediate host in which the parasite undergoes a complex developmental cycle with several rounds of differentiation, proliferation and directed migration. It is well known that the adult tsetse fly shows high resistance to African trypanosomes (especially for *Trypanosoma brucei* sp.), which is reflected by low infection rates in experimental infections (<15%) and natural populations (<1%). Parasites acquired by the fly must adapt and establish in the tsetse fly alimentary tract where they are challenged by the fly innate defense system [1]. Then, parasites migrate upstream into the foregut and proboscis where they have to undergo a complex differentiation. Only a few parasites are able to reach the salivary glands where they attach to the salivary gland epithelial cells and start proliferating vigorously [2, 3]. A part of these attached epimastigotes generate progenitor cells that further develop into the final infective metacyclics that are free-living in the tsetse saliva [4]. At this stage of infection, the *T. brucei* population in the tsetse salivary gland is at high density consisting of both the metacyclics as well as a high number of developing parasites that are tightly attached to the gland epithelial cells. It was recently shown that this parasite infection led to a drastic change in the abundance of major saliva proteins resulting in a less efficient tsetse fly feeding process [5]. This reduced expression of saliva proteins was later confirmed in a transcriptome analysis on trypanosome-infected flies [6]. In our study we used an extensive RNA-seq approach in which we compared the whole transcriptome profiles of different age-matched experimental groups of *T. brucei*-infected tsetse flies i.e. flies containing both salivary gland/midgut infection and flies with only a midgut infection, and of non-infected flies. From this differential gene expression analysis we tried to deduce deeper insights on the local parasite-modulated immune responses, cellular damage and repair mechanisms and detoxification of the salivary gland environment.

With this experimental approach we aimed to address two main questions: i) what is the impact of a *T. brucei* infection in the tsetse salivary glands on different aspects of its biological functioning, and ii) which mechanisms are enabling the fly to tolerate the infection i.e. to control the negative impact of the parasite presence. These results are then summarized in a *T. brucei* - tsetse salivary gland interaction model. Besides, a transcriptome comparison with age-matched ﻿salivary glands﻿ non-infected flies indicated a set of genes with a modified expression in the salivary glands of midgut-only infected flies, so before parasites were present in this tissue.

## Methods

### Tsetse flies infection and salivary glands collection

Male *Glossina morsitans morsitans* (*Gmm*) from the colony at the Institute of Tropical Medicine (Antwerp, Belgium) were used in all experiments [7]. For the infection experiment the pleiomorphic *Trypanosoma brucei brucei* (*Tbb*) AnTAR1, derived from the EATRO 1125 strain [8] was used. This tsetse-trypanosome infection model has already been shown to result in good infection rates in the fly midgut and salivary glands, with high metacyclic parasite densities in the latter [2, 9]. Freshly emerged flies were offered their first blood meal on an anaesthetized mouse showing a parasitaemia of approximately 10^8^ trypanosomes/ml blood with 80% intermediate/stumpy forms. Only fully engorged flies were selected and maintained for four weeks at 26 °C/65% relative humidity and were offered a blood meal three times per week using an artificial membrane feeding system. Twenty-eight days after the infective blood meal, individual flies were evaluated for the presence of metacyclic trypanosomes in their salivary glands by salivation on pre-warmed (37 °C) glass slides (modification of the method of Burtt et al. [10]). Immediately after saliva evaluation, the tsetse flies received a blood meal and were maintained for another 72h before the salivary glands were collected by dissection. Each sample consisted of a pool of 20 salivary glands. Three experimental groups of age-matched tsetse flies with the same feeding regimen but with a different trypanosome-infection status were compared in this study: a) flies harboring a mature trypanosome infection in the salivary glands as well as in the midgut (SG + MG+); b) flies containing only an infection at the midgut level but not at the salivary glands (SG-MG+); c) flies that were never exposed to a trypanosome infection (SG-MG-; non-infected). For each experimental group three independent biological replicates were generated.

### RNA isolation, library construction and sequencing

Total RNA was extracted from samples using RNAqueous®-Micro Kit (Ambion), following manufacture's instructions. Total RNA concentration was quantified by measurement of the 260 nm absorbance with an ND-1000 spectrophotometer (NanoDrop Technologies, Rockland, DE, USA). RNA quality was analyzed by assessing the 260/280 nm and 260/230 absorbance ratios and by using the Agilent RNA 6000 Nano Kit on a Agilent 2100 Bioanalyzer (Agilent Technologies). RNA-Seq libraries were constructed according to the TruSeq® Stranded mRNA Sample Preparation Guide (Illumina, Inc). Briefly, the protocol included purification of the poly-A containing mRNA molecules from 700 ng of total RNA using poly-T oligo attached magnetic beads. Next, the mRNA was fragmented and first strand cDNA was synthesized. During the second cDNA strand synthesis step the RNA template was removed and a replacement strand, incorporating dUTP in place of dTTP was produced generating double-stranded (ds) cDNA. Afterwards, the ds cDNA libraries were 3′ends adenylated, barcoded with Truseq adaptors and PCR enriched. All libraries were, pooled and multiplexed across eight lanes and sequenced on an Illumina HiSeq1500 instrument (performed at the University of Antwerp). To optimize the output and to minimize the confounding effects of lane to lane [11] each library was duplicated (SG-MG+ and SG-MG-) or triplicated (SG + MG+) over different lanes. The libraries were single-end, sequenced for 50 base pairs.

### Reference based annotation and detection of differentially expressed genes

The RNA-seq reads were first filtered based on the quality scores. The *Glossina morsitans* reference genome [12] (assembly GmorY1 was obtained from VectorBase [13] (https://www.vectorbase.org/). During our analysis we observed that a set of genes were missing from the assembly and we added them manually (Additional file 1: Table S1), and used this data set for further analysis. The quality filtered reads were aligned with STAR (v2.3) [14, 15] with optimized parameters concerning the mapped reads and the alignment (outFilterScoreMinOverLread 0.4; outFilterMatchNmin 40; outFilterMatchNminOverLread 0.4; outFilterMismatchNoverLmax 0.05; outFilterType BySJout) and the splice junctions (outSJfilterOverhangMin −1 25 25 25; outSJfilterCountUniqueMin −1 10 10 10; sjdbOverhang 49). Read-mapping statistics were calculated with bam_stat.py included in the RSeQC v2.3.6 software package [16]. The technical replicates BAM files (reads belonging to the same library that were sequenced in different lanes) were sorted, attributed a read group by Picard [17] and merged in one BAM file per sample with SAMtools [18]. Reads were mapped to the *T. brucei* genome (TbruceiTREU927_v6) downloaded from TriTrypDB [19] (http://tritrypdb.org/tritrypdb/) using STAR (v2.3) with default parameters, but the genes expression was not further analyzed for this paper. The raw sequencing reads have been deposed to the Short Read Archive in the BioProject with Accession Number PRJNA327366 ﻿and SRA Study accession number SRP093425.

The Python package HTSeq (v0.5.4) [20], with the intersection-nonempty mode, was used to enumerate the number of reads per transcript to the gene dataset *GmorY1.5* (13 034 gene models) + 3 models added manually (Additional file 1: Table S1). DESeq2 package [21] (R version, 3.2, DESeq2 version, 1.8.2) was used for the differential expression analysis. DESeq2 is part of the Bioconductor set of software packages [22], and uses the R statistical programming language [23]. For the differential expression analysis of transcripts affected by the *Tbb* at salivary gland level we made the following comparisons: 1) flies with a mature *Tbb*-infection (SG + MG+) versus non-infected (SG-MG-) flies and 2) flies with a mature *Tbb*-infection (SG + MG+) versus flies with an established *Tbb* midgut infection only (SG-MG+). Transcripts were considered differentially expressed if showing a *p value* < 0.05 and an adjusted P value lower than 10%. The heat maps were generated with the pheatmap (Pretty Heatmaps) function in the pheatmap package (R version, 3.2, pheatmap version, 1.0.8 [23]).

### Differentially expressed transcripts annotation

The list of differentially expressed transcripts was functionally annotated using several methods. First the VectorBase transcripts annotations were retrieved (GmorY1.5, 13200 predicted transcripts from which 12552 are protein coding and 8001 are hypothetical proteins). Followed by a blastx analysis with the DIAMOND program [24] against the UniProt *Drosophila melanogaster* reference proteome (UP000000803) and *Glossina morsitans morsitans* available sequences (2639). The results were filtered to only retain hits with an E-value <1e^−10^ and a BitScore > 60. Groups of orthologous protein sequences were identified with the OrthoMCL algorithm [25] on the http://www.orthomcl.org/ server. The Gene Ontology (GO) terms were added with Blast2GO [26, 27], using the blastx algorithm and significance threshold of 1 × 10^−06^ to search against *Drosophila* database and NCBI’s non-redundant (NR) protein database. To assess which GO terms were overexpressed relative to the entire transcriptome an enrichment analysis (Fisher's exact test) in Blast2GO was carried out. Further in the text for the not annotated genes we used the name of the *Drosophila* orthologue.

The putative members of *Glossina* innate immunity pathways (Imd, Toll, JAK/STAT) were identified by blastp analysis with the DIAMOND [24] program using Drosophila ﻿orthologues (downloaded from www.flybase.org) against the GmorY1.5 predicted proteins data set. For the obtained *Gmm* orthologues the transcripts were extracted and used in blastx searches on the NCBI Blast (http://blast.ncbi.nlm.nih.gov) to confirm the attributed putative function. The pathways diagrams were created in yEd Graph editor (v 3.14.4).

### qPCR quantitative gene expression analysis for transcriptome validation

A total of 16 genes identified by RNA-seq to be differentially expressed were chosen for real-time quantitative PCR analysis (Additional file 2: Table S2). The total RNA extracted for the RNA-seq library construction (see above) was used for this analysis. Samples were DNAse I treated and afterwards first strand cDNA was reverse transcribed from 700 ng RNA using oligo(dT)_15_ primer and Transcriptor Reverse Transcriptase (Roche), following the manufacturer’s instructions. Real-time quantitative PCR reactions of 20 μl were performed with SensiMIX™ SYBR® No-ROX kit (Bioline) and 0.5 μM of each primer (except *Tsal1*and *Sgp2* that were 0.7 μM). Real-time quantitative PCR reactions were run on a Light Cycler 480 system (Roche Diagnostics). For each condition three replicates were used. To select a set of suitable reference genes, the RNA-seq samples were normalized together in DESeq2 and the normalized read counts were filtered for genes with a relative standard deviation < 6% and normalized read counts >1000. Forty-five genes passed the filters, from which nine were selected based on the presence of orthologues or GO terms. Primers were designed and PCR efficiency and amplification specificity was determined. The final data set included six genes identified by RNA-seq data analysis and three other ones commonly used (Additional file 2: Table S2). qPCR results were analyzed using the BioGazelle qbase plus 1.5 software to evaluate reference gene stability and to obtain normalized values for the tested genes in the different salivary gland tissue samples.

## Results and discussion

### Sequencing and mapping results

**Fig. 1:**
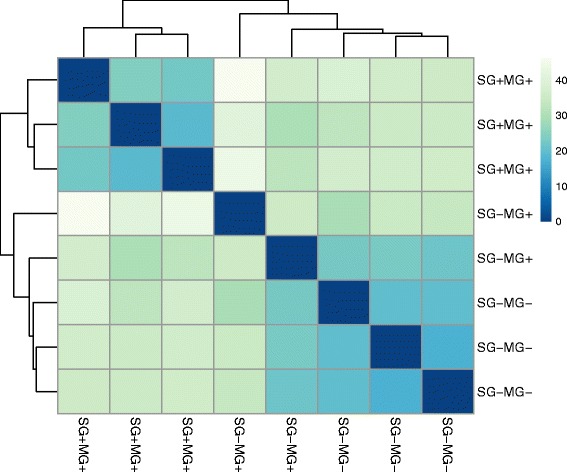
Heat map showing the Euclidean distances between the samples as calculated from the regularized log transformation. MG: midgut; SG: salivary glands. The trypanosome infection status of the respective tissue is indicated by – or +

The Illumina RNA-seq technology was used to characterize *Tbb*-infected and non-infected salivary glands whole transcriptomes. Single-end RNA-seq libraries were constructed starting from pools of 20 salivary glands. After sequencing a total of 369 million high quality raw reads were obtained across all nine samples, ranging from 44 to 58 million raw reads for the SG + MG+ replicates, from 25 to 28 million raw reads for the SG-MG+ replicates and from 28 to 55 million raw reads for SG-MG- replicates (Table 1). More than 80% of the raw reads were successfully uniquely mapped onto the *G. morsitans* genome for the uninfected salivary glands samples (SG-MG+ and SG-MG-) and 60% of the raw reads for the *Tbb*-infected glands (SG + MG+). For the *Tbb*-infected glands samples about 21% of the reads mapped to the *T. brucei* genome (Table 1). Each condition was represented in the final dataset by three biological replicates, except for the SG-MG+ series where one replicate, R3 contained 1.94% reads that unexpectedly mapped onto the *T. brucei* genome, probably because of one fly from the pooled sample had developed a salivary gland infection during the 72 hours-period between the saliva evaluation and the tissue sampling for RNA extraction. This replicate was removed from further analysis. Data quality was evaluated by Pearson’s *r* value determining the correlation between replicates (Table 1) and by sample clustering of the Euclidean distances between all eight libraries, with the SG + MG+ samples grouped in a separate cluster (Fig. 1). A transcription signal was detected for approximately 58% of the known *G. morsitans* genes, where we considered a gene as being expressed if read coverage was higher than 1x (Table 1).

**Table 1:** Summary result of mapped reads

Condition^a^	BRep^b^	Total reads^c^	UMR to *Gmm* ^d^	%^e^	No. Trans.^f^	%^g^	MR to *Tbb* ^h^	%^i^	PCC^j^	
SG + MG+	R1	57329317	38764267	67.62	7436	57.36	9033083	15.75	0.993	R1 vs R2
	R2	58422283	36875950	63.12	7451	57.48	12728367	21.78	0.990	R2 vs R3
	R3	44939446	27147298	60.41	7537	58.14	11826098	26.31	0.991	R1 vs R3
SG-MG+	R1	28963806	23991706	82.83	7570	58.40	83271	0.32	0.98	R1 vs R2
	R2	25976974	20904134	80.47	7499	57.85	184360	0.60	0.98	R2 vs R3
	R3	22656455	17897012	79.99	7420	57.24	439615	1.94	0.98	R1 vs R3
SG-MG-	R1	48197948	39337062	81.62	7554	58.27	116495	0.24	0.989	R1 vs R2
	R2	55170655	44055207	79.85	7522	58.03	124863	0.22	0.990	R2 vs R3
	R3	28013655	22488365	80.28	7482	57.72	69501	0.22	0.989	R1 vs R3

^a^MG midgut, SG salivary glands. The trypanosome infection status of the respective tissue is indicated by – or +

^b^ *BRep* Biological Replicate

^c^The total numbers of raw reads obtained after sequencing

^d^The number of Uniquely Mapped Reads to *Glossina morsitans* (assembly GmorY1)

^e^The percentage of UMR reported to the total﻿ number of reads

^f^Number of expressed transcripts, a transcript was considered expressed if the normalized reads coverage was higher than 1x

^g^The percentage of 6 reported to the gene dataset *GmorY1.5*

^h^The number of Mapped Reads to *Trypanosoma brucei* (assembly TbruceiTREU927_v6.0)

^i^The percentage of MR reported to the total number of reads

^j^Pearson Correlation Coefficient between replicates

### Differentially expressed genes in the tsetse salivary glands of *Tbb*-infected flies

**Fig. 2:**
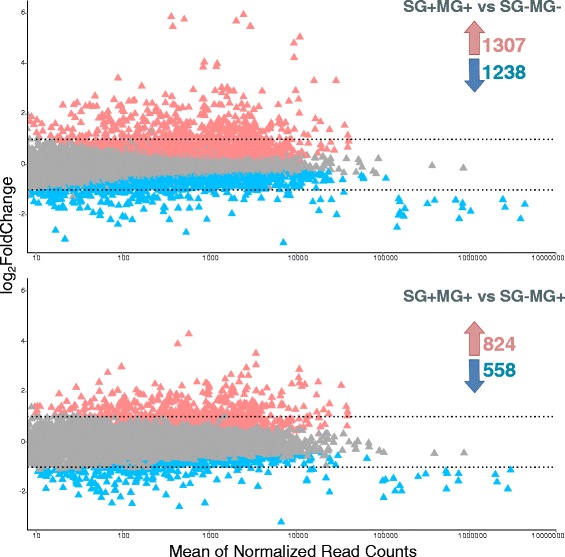
Differentially expressed transcripts between the experimental conditions. *Red* and *blue triangles* indicate significance at a 10% adjusted p-value, upregulation and downregulation respectively. *Grey triangles* indicate transcripts that showed no change. ﻿MG: midgut; SG: salivary glands. The trypanosome infection status of the respective tissue is indicated by – or +

We assessed the impact of a trypanosome infection on the salivary glands gene expression by performing two transcriptional comparisons: 1) flies with a mature *Tbb*-infection in the salivary glands (SG + MG+) versus non-infected flies (SG-MG-) and 2) flies with a mature *Tbb*-infection in the salivary glands (SG + MG+) versus flies with only an established *Tbb* midgut infection (SG-MG+). All flies in the different groups had the same age and feeding history. Transcripts that scored a *p* value < 0.05 and an adjusted *p* value < 10% were considered differentially expressed. In the SG + MG+ versus SG-MG- comparison 1307 and 1238 transcripts were identified to be respectively induced (with 30.2% >2-fold expression increase) and repressed (with 15.6% > 2-fold expression decrease), linked to the parasite infection in the fly (Fig. 2; and Additional file 3: Table S3). In the SG + MG+ versus SG-MG+ comparison 824 salivary gland transcripts were found to be induced (with 41.7% >2-fold increase) and 558 repressed (with 30% > 2-fold decrease) (Fig. 2; and Additional file 4: Table S4). This differential gene expression in the salivary glands could be attributed to the parasite infection of the tissue.

**Fig. 3:**
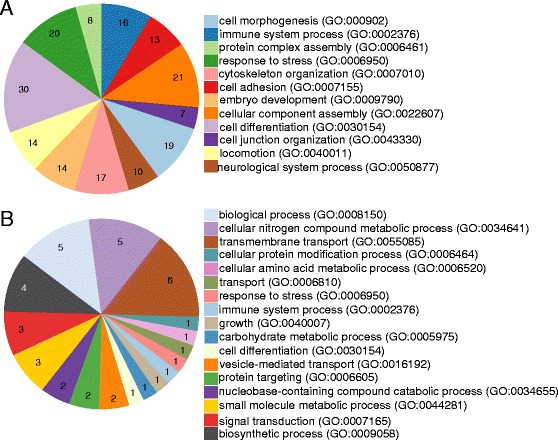
**a** Gene Ontology enrichment analysis for biological process of up-regulated transcripts. The transcripts included had an expression value higher than 2 fold change in both comparisons. The GO terms were slimmed prior to the analysis and were considered significant for a false discovery rate of 0.01. A total of 290 transcripts were used in the analysis from which 217 had a GO term annotation. The number of transcripts with a GO term is indicated in the corresponding pie slice. **b** Classification of downregulated transcripts using Gene Ontology. The transcripts included had an expression value lower than 2 fold change in both comparisons. A total of 91 transcripts were used in the classification from which 56 had a GO annotation. The GO terms were slimmed prior to the analysis. The number of transcripts with a GO term is indicated in the corresponding pie slice

In our functional analysis of the RNA-seq data we focused on a core group of 1214 transcripts that showed a similar differential expression pattern in both salivary glands transcriptome comparisons (Additional file 5: Figure S1 and Additional file 6: Table S5). This group of genes could be considered as differentially expressed due to the *Tbb* infection in the salivary glands. The enrichment analysis of GO categories was conducted only for the >2-fold differentially expressed transcripts. For the upregulated transcripts, GO-categories like cell differentiation, response to stress, cytoskeleton organization, immune system processes and many others (Fig. 3a) were enriched. For the downregulated transcripts, a classification of the GO term biological process is presented in Fig. 3b. No significantly enriched GO-categories could be identified here.

To validate the RNA-seq data we performed a real-time quantitative RT-PCR on a selection of 16 genes that came out as differentially expressed in the RNA-seq data analysis. Fold-changes in gene expression were derived by the comparative C^t^ method, using *rp49* (GMOY001799) and GMOY006676 as reference genes. The two genes were identified as being the most stable ones based on a geNorm analysis. The results confirmed the transcriptional expression changes (up- or down-regulated) and the correlation between the two methods by Pearson’s *r* value of 0.930 for SG + MG+ versus SG-MG- and 0.937 for SG + MG+ versus SG-MG+ as shown in Additional file 2: Table S2.

### Detailed sequence analysis and function prediction of differentially expressed transcripts in the salivary glands of *Tbb*-infected flies

The data analysis revealed a complex change in transcript expression in the salivary glands as a result of the parasite infection. Sequence analysis and function prediction indicated that many different biological pathways and processes are being affected like the blood feeding process, tsetse innate immune system, cellular detoxification processes, cytoskeleton assembly, cell adhesion, and many others. In the subsequent sections, we describe the analysis on a selection of these genes based on their differential expression and putative function.

#### Genes encoding for the major tsetse saliva proteins

**Fig. 4:**
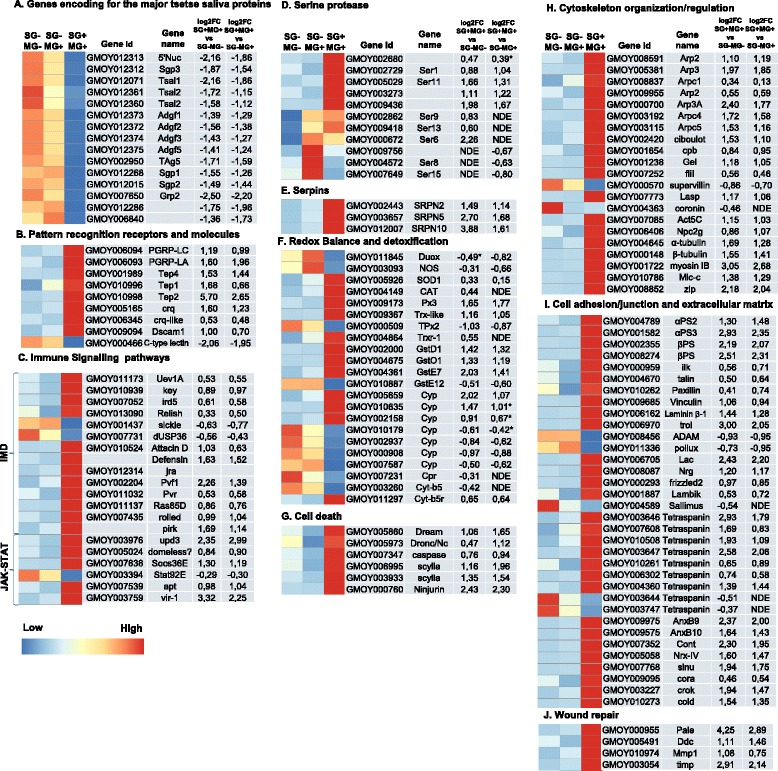
**a-j** Graphical representation of transcripts expression in different functional category. The heat maps were obtained by plotting the mean of normalized read counts (scaled by row) in the three infection conditions. Colors display z-scores from −1 (low expression: *dark blue*) to 1 (high expression: *red*) for normalized gene expression values. ﻿﻿﻿MG: midgut; SG: salivary glands. The trypanosome infection status of the respective tissue is indicated by – or +. FC: Fold Change﻿﻿; NDE: not differentially expressed

Several genes encoding the major tsetse saliva proteins were significantly compromised by *Tbb* presence in the glands (Fig. 4a). This concerns genes that code for the salivary apyrases *5'Nuc* (GMOY012313) and *Gmm* salivary gland protein 3 (GMOY012312), the nucleic acid binding tsetse salivary gland proteins *Tsal1* (GMOY012071) and *Tsal2* (GMOY012361, GMOY012360), adenosine deaminase growth factors *Adgf1* (GMOY012373), *Adgf2* (GMOY012372), *Adgf3* (GMOY012374), *Adgf5* (GMOY012375); and the tsetse Antigen5 *TAg5* allergen (GMOY002950). Other salivary genes encoding the glycine/glutamate-rich protein (*Sgp1,* GMOY012268), the proline-rich protein (*Sgp2*, GMOY012015) and the glycine-rich protein 2 (GMOY007650) were more than 2-fold downregulated. This downregulation of the major salivary genes due to the presence of a *Tbb* infection in the tsetse salivary glands is in accordance with previous reported findings [5, 6]. In addition, two other hypothetical secreted peptides (GMOY012286, GMOY006840) that were previously described in the *Gmm* sialome [28], also showed a highly compromised expression. In contrast, the expression of the gene coding for *TTI* (GMOY012244), a major anti-thrombine saliva peptide in *Gmm* [29, 30], was not significantly affected, and showed high variability between replicates similar as described in [6]. These results clearly confirm that the presence of a *T. brucei* infection in the tsetse fly salivary glands has a strong negative impact on the biological functioning of the tissue reflected mainly by the high decrease in expression of a set of genes coding for the major anti-haemostatic proteins in the tsetse saliva. As this arsenal of saliva proteins is essential to facilitate the tsetse blood feeding and digestion process, the presence of a trypanosome infection compromises significantly the tsetse probing and feeding efficiency as demonstrated by [5].

#### Immunity-related genes: pattern recognition and signaling pathways

Immune reactions are initiated when microbial surface molecules are recognized as “non-self” by pattern recognition receptors (PRRs) that bind to pathogen-associated molecules. Two peptidoglycan recognition proteins associated with the Imd-signaling pathway showed an increased transcript level upon trypanosome infection in the salivary gland: *PGRP-LC* (GMOY006094) and *PGRP-LA* (GMOY006093) (Fig. 4b). Another family of PRR-associated proteins that showed an increased expression in the trypanosome-infected glands are the thioester-containing proteins (TEPs). TEPs show high similarity to mammalian complement C3 and are involved in innate immunity in arthropods [31–33]. The TEP protein repertoire was shown to be involved in the insect defense responses against different types of microbes like the binding and killing of *Plasmodium berghei* ookinetes [32], the clearing of bacteria [33, 34] and *Candida albicans* [33, 35] via phagocytosis. Recently, the TEP-Macroglobulin complement-related (Mcr) protein from *Aedes aegypti* was reported to control the dengue virus infection by induction of antimicrobial peptides [36]. In the *G. morsitans* genome, the TEP family is represented by four genes: one Mcr *Tep4* (GMOY001989) and three insect TEPs: *Tep1* (GMOY010996), *Tep2* (GMOY010998) and *Tep3* (GMOY008955). In the *Tbb*-infected glands *Tep1*, *Tep2* and *Tep4* showed an increased expression with *Tep2* being highly upregulated (Fig. 4b) 52-fold for SG + MG+ versus SG-MG- and 6-fold for SG + MG+ versus SG-MG+, in agreement with [6]. The strong upregulation of the different *Teps* (especially *Tep2)* in the salivary glands in the presence of the trypanosome infection suggests a possible role of this protein family in the interaction with the *Tbb* parasite population in the tissue. Moreover, the high similarity of the predicted Tep4 protein with the DmTep6-Mcr protein in *Drosophila* could indicate a similar role of this protein as described for *Drosophila* in the formation and maintenance of the septate junctions in the epithelial cell lining in the tsetse salivary glands [37, 38]. Indeed, the tight attachment of the trypanosome flagellum to this epithelial lining [39] in the infected glands could possibly cause damage to the septate junctions. The upregulation of *Tep4* in the glands would therefore be necessary to ensure the structural integrity of the tsetse salivary gland epithelium thereby controlling the parasite-caused epithelial barrier damage.

Other recognition proteins encoding genes that were upregulated by trypanosome infection in the salivary glands include the class B scavenger receptor *croquemort* (GMOY005165), a *croquemort-like receptor* (GMOY006345) and *Dscam1* (GMOY009094). Croquemort has been demonstrated in *Drosophila* to be involved in the clearance process of apoptotic cells [40] and autophagic cell death [41], and in the phagocytosis of Gram positive bacteria [42]. *Dscam1* is a mosaic protein that can form a complex set of pathogen-specific splice repertoires and has been shown in *Anopheles* mosquitoes to be involved in the defense against bacteria and *Plasmodium* parasites [43, 44]. Surprisingly, the C-type lectin (CTL) (GMOY000466) that is present as a soluble factor in saliva [28], was highly downregulated (>4-fold) similarly as was described for the major secreted saliva protein genes, a similar downregulation of two C-type lectins had been reported in [6]. CTLs comprise a large superfamily of proteins, which recognize a diverse range of ligands, and are defined by the presence of at least one C-type lectin-like domain. Carbohydrate/CTL interactions occur on cell surfaces, in the extracellular matrix (ECM), or on soluble secreted glycoproteins and may mediate processes such as cell adhesion, cell/cell interactions, glycoprotein turnover, and pathogen recognition leading to innate immune responses. In vertebrates, CTLs are important components of cellular as well as humoral innate immune responses to several classes of microbe and recognize and trigger cellular responses to dead cells [45–47]. Several immune functions have been proposed for insect CTLs, including activation of the prophenol oxidase cascade, hemocyte-mediated encapsulation, nodule formation, and opsonisation. In mosquitoes, CTLs were demonstrated to play a role in the antibacterial defense as well as protective agonists on the *Plasmodium* parasite development in the mosquito gut [45, 48]. It is clear that the presence of the *Tbb* parasite in the salivary glands alters the expression of some genes encoding important pathogen recognition proteins. It can be assumed that this result in some downstream effects contributing to the control of the trypanosome infection in the tsetse fly salivary glands and its related tissue damage but this remains to be experimentally elucidated.﻿

﻿Insects possess three intracellular signaling pathways for microbe’s recognition and immune response: Imd (Immune deficiency), Toll, and JAK/STAT pathway. Activation of these pathways can result in the production of different pathogen-effector molecules such as various antimicrobial peptides (AMPs). Analysis of our RNA-seq data revealed that the trypanosome-infected salivary glands are enriched for several transcripts that are linked with the Imd- and JAK/STAT pathway (Fig. 4c, Additional file 7: Figure S3 and Additional file 8: Figure S4). This indicates that the salivary gland tissue is mounting a local immune reaction in response to the present parasite infection. Several components involved in Imd activation and downstream induction of antimicrobial peptides showed an increased expression level in infected glands, such as *Uev1A* (GMOY011173) and the IKK complex constituents *kenny* (GMOY010939) and *immune response deficient 5* (GMOY007052). The key-transcription factor *Relish* (GMOY013090) was moderately upregulated. Furthermore, orthologues genes from the fruit fly genome for the negative regulator *pirk* (not annotated assembly GmorY1, see Additional file 1: Table S1), the *Pvr* receptor (GMOY011032) and ligand *Pvf1* (GMOY002204) were found to be upregulated indicating a regulatory control of the Ras/MAPK signaling pathway for the Imd pathway in the salivary glands [49]. For the downstream antimicrobial effector peptides, the genes coding for *Defensin* (not annotated assembly GmorY1, see Additional file 1: Table S1) and *Attacin D* (GMOY010524) were found upregulated in the *Tbb*-infected glands, *Attacin D* only in SG + MG+ vs SG-MG- (Fig. 4c). The observed upregulated AMP expression in this data set is consistent with the described role of antimicrobial defense peptides against a *Tbb* infection in the tsetse alimentary tract. Indeed, in flies with established midgut infection, *Defensin*, *Attacin* and *Cecropin* were detected in fat body and proventriculus [50–52], as well in the haemolymph [53]. Moreover, *Attacin* together with *Cecropin* were shown to be upregulated in the midgut of self-cleared flies [54] in a *Relish* dependent manner, indicating the importance of the Imd pathway in parasite control in the tsetse midgut [55].

Along with the Imd pathway, expression of various constituents of the JAK/STAT pathway was affected by the trypanosome presence in the salivary gland. The expression of *upd3* (GMOY003976), a cytokine involved in the dimerization of the membrane receptor *domeless,* was increased. The expression of a cytokine receptor (GMOY005024), a possible *domeless* orthologue was upregulated as well. Two negative regulators of the cascade JAK/STAT cascade, *Socs36E* (GMOY007838) and *apontic* (GMOY007539), were  also upregulated. In contrast, the signal transducer and transcription activator *Stat92E* (GMOY003394) was moderately downregulated only in the SG + MG+ versus SG-MG+ with an adjusted *p-value* <10%. The *vir-1* gene, encoding the vir-1 effector molecule, was found to be highly upregulated (GMOY003759) (Fig. 4c). *Stat92E* expression was not changed  in a trypanosome-infected midgut [54] plus *domeless* expression was not  affected in parasite challenged versus unchallenged *Gmm* flies [56]. So far, taking our RNA-seq data and previously reported results into consideration, it remains unclear whether the JAK/STAT mediated immune response is actually involved in the control of the trypanosome infection in the tsetse salivary glands. Similar to mammals, the JAK/STAT pathway in *Drosophila* and mosquitoes is described as a key antiviral player. In fruit flies, the JAK/STAT pathway has been implicated in the control of *Drosophila* C virus (DCV) infection [57]. The involvement of the JAK/STAT pathway in pathogen control in the mosquito-pathogen interaction for e.g. *Anopheles gambiae* where this pathway is activated in response to bacterial challenge [58] and viral load (reviewed [59]), and was shown to regulate nitric oxide synthase (NOS) expression in a late anti-plasmodial response phase [60]. In the fruit fly, this pathway is activated in response to bacterial challenge and was demonstrated to control the expression of the thioester-containing protein (*Tep1*) [61]. Vir-1 protein has been identified in the tsetse sialome [28]. *Vir-1* function is unknown in fruit fly but it has been associated with the JAK/STAT pathway and considered to be virus induced [57].

For the Toll-signaling pathway only two genes, *tube* (GMOY007350) and *dorsal* (GMOY004479) showed moderate expression changes and only in the of SG + MG+ vs SG-MG- comparison (Additional file 9: Figure S5). This indicates that this pathway is not activated significantly by the trypanosome infection in the fly.

#### Serine proteases and serpins

Serine proteases (SPs) and serine proteases homologues (SPHs) belong to a large family of proteins in insects with a variety of important roles in e.g. digestion and cellular/humoral immunity [62, 63]. Usually, SPs are produced as inactive zymogens that have to be proteolytically cleaved to obtain the active conformation. Many SPs and SPHs have domains or other structural additions with key roles for protein-protein interaction [64]. One of the main group of regulatory modules of SPs is the clip domain family. In our data set, the expression of eleven SPs and SPHs were found to be affected by the parasite infection. Two clip-domain serine proteases - GMOY003273 and *Ser11* (GMOY005029), GMOY009436 (coding for a sushi domain SPH) and *Ser1* (GMOY002729) were upregulated in both transcriptional comparisons (Fig. 4d). Clip-domain SPs are associated with innate immune responses in invertebrates, being essential components of the extracellular signaling cascade. Sushi domain SPs are known to be involved in recognition processes with important roles in regulating the complement system. Three SPs were only found to be differentially expressed in the SG + MG+ versus SG-MG- comparison: *Ser6* (GMOY000672) with a more than 4-fold increase, *Ser9* (GMOY002862) and *Ser13* (GMOY009418) both with a moderate increase.

Serpins are the largest family of serine protease inhibitors and were shown to be key regulators of innate immune reactions, activation of pro-phenoloxidase and hence melanisation, proteolytic activation of Toll pathway and activation of the complement like-system by proteolytic cleavage of thioester-containing proteins (reviewed in [65]). In the mosquito-*Plasmodium* interaction, an immune-responsive serpin was demonstrated to control the parasite population in the gut and salivary glands of the mosquito [66, 67]. Three genes coding for serpins where found to be upregulated in both comparisons: *SRPN2* (GMOY002443), *SRPN5* (GMOY003657), *SRPN10* (GMOY012007) (Fig. 4e), high upregulation of two of these serpins was also documented in [6]. Recently, tsetse *SRPN10* was shown to have a role in the inactivation of the complement system by inhibiting the activity of cascade activators present in ingested blood meal [68]. Further studies will be needed to clarify the role of the high expression of the serine protease and serpins in the trypanosome-infected salivary gland tissue.

#### Redox balance and detoxification

Trypanosome infection in the tsetse fly salivary glands resulted in the increased expression of a series of genes/enzymes involved in detoxification of the cellular environment and the downregulation of some key actors responsible for generation of reactive oxygen species (ROS)/reactive nitrogen species (RNS) (Fig. 4f) i.e. the nitric oxide synthase encoding gene (GMOY003093) and the *Dual oxidase* (GMOY011845). Detoxification enzymes protect the host cells from oxidative damage. A set of genes coding for different detoxifying enzymes are found to be significantly upregulated in the trypanosome infected glands: *Peroxidase 3* (GMOY009173), thioredoxin-like encoding gene (GMOY009367), glutathione S transferases (GTSs) *GstD1* (GMOY002000), *GstO1* (GMOY004675), *GstE7* (GMOY004361). A similar observation of elevated expression levels of genes coding for detoxification enzymes has been reported in the tsetse midgut in response to trypanosome infection or blood feeding [54, 69]. In insects, besides a central role in the metabolism of xenobiotic compounds like insecticides, GTSs are also involved in various biological processes including protection against oxidative stress [70], bacterial infection immune response [71, 72], and preservation of redox status in relation with vectorial capacity [73]. In the case of the trypanosome-tsetse fly interaction, GSTs role in the midgut was suggested to be protective in response to the heme in the blood meal [74].

The expression of seven genes coding for proteins that belong to the super-family of detoxification enzymes, the P450-cytochromes (CYPs), as well as *Cytochrome P450 reductase* (GMOY007231), *Cytochrome b5* (GMOY003260) and *Cytochrome-b5 reductase* (GMOY011297) were also affected (up and downregulated) by the trypanosome infection in the glands. The expression of two CYPs (GMOY005659, GMOY010635) was highly increased (above 2-fold). CYPs are known to be involved in the insect metabolism, development and detoxification. They metabolize endogenous compounds like steroids and lipids and exogenous compounds like insecticides [75]. It has been demonstrated that the transcription of these genes is regulated by the presence of several pathogens, including malaria parasites in the mosquito *A. gambiae* [76].

#### Cell death

Genes related to apoptotic process showed to be affected by the parasite in the salivary glands (Fig. 4g). Three caspases were upregulated: *Dream* (GMOY005860*), Dronc*/*Nc* (GMOY005973) and GMOY007347, a homolog of Death related ICE-like caspase in fruit fly. The two already mentioned scavenger receptors *croquemort* (GMOY005165) and *croquemort-like* (GMOY006345) and two transcripts encoding for *scylla* (GMOY006995; GMOY003933), a RTP801-like mammalian regulator of apoptosis [77], were upregulated as well. *Ninjurin* (GMOY000760), a transmembrane protein, associated with response in septic injury and induction of cell death [78] showed a more than 5 fold increase.

#### Cytoskeleton organization/regulation; cell repair

The expression of a large group genes related to cytoskeleton dynamics is significantly increased, comprising actin and actin-related proteins, tubulins, myosins among others (Fig. 4g).

The Arp2/3 complex composed of seven polypeptides is a multifunctional organizer controlling polymerization, elongation and establishment of actin-filament networks [79]. All seven subunits were upregulated in the trypanosome-infected glands: *Arp2* (GMOY008591), *Arp3* (GMOY005381), *Arpc1/sop2* (GMOY008837), *Arpc2* (GMOY009955), *Arpc3* (GMOY000700), *Arpc4* (GMOY003192), *Arpc5* (GMOY003115). An increased expression of actin filament regulators was also observed: *ciboulot* (GMOY002420) - promoter of actin assembly at filament barbed ends [80] and the F-actin capping protein subunit beta *cpb* (GMOY001654), a terminator of barbed end elongation. Three actin binding proteins from the villin/gelsolin family were differentially expressed, *Gelsolin* (GMOY001238) caps and severs the barbed end of actin filaments; *flightless-1* (GMOY007252), *supervillin* (GMOY000570). *Lasp* (GMOY007773) an actin binding protein that interacts with the integrin *myospheroid* in hub cells to anchor the stem cell niche [81] was upregulated . *Coronin* (GMOY004363), a direct inhibitor of Arp2/3 complex was moderately downregulated. The cytoplasmic *Actin 5C* (GMOY007085) and the homologue of AgMDL1 *Npc2g* (GMOY006406) were found to be upregulated in the *Tbb*-infected glands. The *A. gambiae Actin 5C* was described recently to have a new function as an extracellular pathogen recognition factor involved in antibacterial defense by interaction with the extracellular immune factor AgMDL1. This way the actin plays a role as a *Plasmodium* antagonist, limiting parasite infection in the gut [82]. The presence of the *T. brucei* parasites in the salivary glands induced a more than 3-fold increase expression of *α-tubulin* (GMOY004645) and *β-tubulin-1* (GMOY000148). A relationship between tubulins and P450 cytochromes was described during *A. gambiae* immune response to *P. berghei* invasion [75]. Three myosins were strongly upregulated in the *T. brucei* infected glands: *Myosin IB* (GMOY001722), *Myosin light chain cytoplasmic* (GMOY010786) and *zipper* (GMOY008852).

#### Cell adhesion/junction and extracellular matrix

Integrins are alpha/beta heterodimeric cell-surface receptors that act as a docking site, linking the extracellular matrix molecules to the intracellular cytoskeleton [83]. In conjunction with mediated cell-adhesion function, integrin activation triggers a wide variety of signaling events within the cell, regulating actin cytoskeletal rearrangements, cell morphology, gene expression, cell proliferation and survival [84]. Three integrin genes were upregulated in infected salivary gland, corresponding to alpha subunit *αPS2* (GMOY004789 + GMOY004790), and *αPS3* (GMOY001582) and beta subunit *βPS* (GMOY002355; GMOY008274), with an upregulation of *αPS3* and *βPS* of more than 4-fold (Fig. 4i). Four focal adhesion proteins involved in establishing and maintaining the integrin-cytoskeleton linkage, *Integrin linked kinase* (GMOY000959), *talin* (GMOY004670), *Paxillin* (GMOY010262) and *Vinculin* (GMOY009685) showed a moderate increase. Four integrin ligands were also differentially expressed in the trypanosome-infected glands: the ECM protein *Laminin subunit beta-1* (GMOY006162) and *trol* (GMOY006970), a highly conserved basement membrane-specific heparan sulfate proteoglycan, were upregulated while ADAM metallopeptidase with thrombospondin type 1 motif A (GMOY008456) and *pollux* (GMOY011336) were moderately decreased.

Tetraspanins known as ‘molecular facilitators’, have the ability to associate with integrins, immunoglobulin superfamily proteins, signaling receptors and enzymes forming tetraspanin-enrichened micro domains on the cell surface, regulating in this way many biological process including adhesion, morphology, motility, and proliferation. From the seventeen putative tetraspanins identified in the *Gmm* genome [85], ten genes were found to be differentially expressed in trypanosome-infected glands. Four highly upregulated genes: GMOY003646, *GmTsp5* (GMOY007608), *GmTsp7* (GMOY010508), *GmTsp2* (GMOY003647); four moderately upregulated genes: *GmTsp8* (GMOY004352), *GmTsp39D* (GMOY010261), GMOY006302 and GMOY004360. Two genes were found moderately downregulated and only in the SG + MG+ versus SG-MG- comparison: *GmTsp42Ek* (GMOY003644) and *GmTsp4* (GMOY003747). Tetraspanins induction was observed upon Dengue virus infection in *A. aegypti* salivary gland transcriptome [86]. Two annexin encoding genes were highly upregulated: *Annexin IX* (GMOY009975) and *Annexin X* (GMOY009575). Annexins are scaffolding proteins with the property of binding and holding together biological structures such as membranes. They have been shown to play a role in anticoagulation, endo- and exocytosis, cell adhesion [87], receptor-mediated pathogen uptake [88] and many others. In insects, annexins are considered to be represented by three specific classes (IX, X and XI) [89], although there might be variations between species. In mosquito, the addition of antibodies against recombinant annexins in the blood meal impaired parasite development, suggesting a facilitating role during the midgut epithelium invasion by the *Plasmodium* parasite.

Septate junctions (SJs) are intercellular junctions specific to the invertebrate epithelial cells, displaying a unique ladder-like morphology and forming a paracellular barrier [90]. So far, in *Drosophila* more than 20 genes have been described with a function in the establishment and maintenance of SJs (reviewed in [38]). We identified a series of transcripts encoding for SJs proteins as being upregulated in trypanosome-infected glands including cell adhesion molecules *Contactin* (GMOY007352), *Neuroglian* (GMOY008087), *Neurexin IV* (GMOY005058) and *Lachesin* (GMOY006705); a transmembrane protein *sinuous* (GMOY007768); a cytoplasmic protein *coracle* (GMOY009095) and *crooked* (GMOY003227) and *coiled* (GMOY010273) coding for proteins required for SJ assembly. Similar results were reported in [6] where the GO category septate junction assembly, (counting 11 genes) was found to be enriched in trypanosome infected salivary glands. Recently, two studies showed that the macroglobulin complement-related (Mcr) protein is also a core component of the SJ being essential for the formation and organization of these structure [37, 38]. As already described before, the expression of the gene coding for this Mcr protein (*Tep4*) was increased in infected glands.

### Epithelial repair and wound healing of salivary gland cells/tissue after *T. brucei* colonization

The high upregulation of *Pale* (GMOY000955) gene encoding for tyrosine hydroxylase (syn. tyrosine 3-monooxygenase) and *Ddc* (GMOY005491) gene encoding for dopa decarboxylase, two key enzymes in the biosynthesis pathway of melanin and responsible for the formation of Dopa or Dopamine precursors respectively, provides signs of a wound healing process in *Tbb*-infected glands (Fig. 4j). In the tsetse genome we could identify two genes encoding for pro-phenoloxidase (GMOY010728 and GMOY010972), none of them showing any expression in our samples, so an activation of the melanization cascade in the salivary gland tissue is less probable. Recently, overexpression of *Pale* and *Ddc* was recently associated with pupal melanization in *Spodoptera exigua* [91]. Moreover it was shown that these two genes are activated at the site of aseptic injury and related with wound healing [92].

The gene encoding for the matrix metalloproteinase 1 (Mmp1) (GMOY010974) required in the epidermis to facilitate re-epithelization [93] was found upregulated. Moreover, the *Mmp1* regulator the tissue inhibitor of metalloproteinase (*Timp*) (GMOY003054) was upregulated, more than seven fold changes (Fig. 4j).

### Anticipatory response of the salivary gland environment before *T. brucei* colonization

A set of genes was found upregulated in the non-infected salivary glands as a result of the parasite presence in the tsetse midgut (Fig. 4) suggesting an anticipatory response.

To analyze this in more detail, a multi-group transcriptional comparison between midgut-infected flies (SG + MG+ and SG-MG+) versus non-infected (SG-MG-) flies was performed. A total of 526 genes were considered as differentially expressed. Their normalized read counts were extracted and a clustered heat map was generated (Additional file 10: Figure S2). Based on their expression profile these genes could be grouped in three main clusters (G1, G2 and G3) and are listed in the Additional file 11: Table S6. Group G2 included 101 transcripts that showed an increased expression in SG-MG+ samples, similar with the one of *Tbb*-infected samples (SG + MG+). Although, no significantly enriched GO terms were identified; for the molecular function category terms like GTPase activity, pyrophosphatase activity and RNA binding were vastly present. Furthermore, three serine protease (*Ser6*, *Ser13* and *Ser9*) (Fig. 4d), a lectin (GMOY009521), an autophagy-related 3 gene (GMOY005990), a *chemosensory protein 4* (GMOY010874) were present as well. These significant expression changes of a set of genes in the salivary gland by a midgut-only trypanosome infection is interesting as it suggests an anticipatory response in the non-infected salivary glands. This implies that the trypanosome midgut infection modulates a specific local gene expression in the neighboring salivary gland tissue by means of a tissue-to-tissue immune-related communication. The latter was recently demonstrated for *Drosophila﻿,* where a local infection in the gut elicits also an immune response in the fly fat body, with hemocytes serving as signaling relay [94, 95]. Additional studies using more biological replicates, different time points of infection and including other trypanosome transited tissues will allow to document in more detail this putative midgut infection-induced anticipatory response in the tsetse salivary glands.

### Tsetse salivary gland tolerance of *T. brucei* infection

The *T. brucei* parasite undergoes an obligatory and complex developmental journey through the tsetse alimentary tract with a final phase in the salivary glands. The *T. brucei* population in the tsetse salivary gland is at high density consisting of both free-living metacyclics as well as a high number of developing parasites that are tightly attached to the gland epithelial cells. This parasite-epithelial cell junctional complex is described as ‘hemi-desmosome’-like attachment plaques, adhering the flagellar outgrowths of the epimastigote parasite to the apical surface of the insect epithelial cells [96]. In order to have a better insight on the impact of this densely packed *T. brucei*-infection on the salivary gland tissue and its functioning, we performed an extensive RNA-seq based whole salivary gland transcriptome comparison of *T. brucei*-infected versus non-infected flies.

**Fig. 5:**
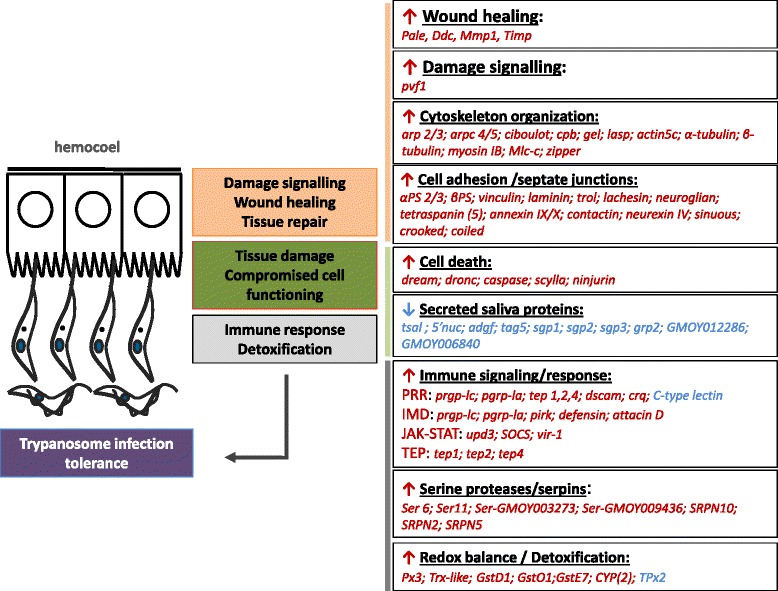
Overview of the major gene transcript impact of *T. brucei* infection in the salivary glands. Only the transcripts that showed a ≥ 2 fold differential expression are presented in the figure. *Red*: increased expression; *Blue*: decreased expression

The previously reported reduced expression of major secreted saliva proteins that are essential in the anti-hemostatic activity of the tsetse saliva [5, 6] was confirmed by our analysis (Fig. 5). This clearly indicates that the parasite infection hampers significantly the normal functioning of the salivary glands resulting in a strong suppression of the continuous production and secretion of an arsenal of proteins that are essential for the blood feeding tsetse fly to feed and digest in an efficient way. So far, besides a longer feeding time required to obtain a full blood meal, it is not documented whether this parasite salivary gland infection also negatively impacts the tsetse fly reproduction and longevity. However, our RNA-seq data unambiguously reveals that the parasite infection has a serious impact on the salivary gland epithelial structure and the gland microenvironment. Indeed, expression of several genes that are linked with tissue damage, cell death, cell repair and cytoskeleton organization is significantly increased in the trypanosome-infected glands, in line with the report of [6]. Moreover, several genes coding for key enzymes in maintaining the redox balance and in detoxification processes are also upregulated in these glands (Fig. 5). In addition, an activated Imd-related immune response with attacin and defensin as main effectors was observed, as well as a significant upregulation of several serine proteases and/or serpins. Activation of all these suggests a strong local tissue response in trying to keep the local cell damage and the parasite infection under control i.e. to tolerate the infection. Insects have the ability to elicit a set of responses to buffer the negative impact on the insect’s fitness of pathogen-induced damage when infection has occurred, without eliminating the pathogen [97, 98]. By this tolerance response, the infection is maintained at a level acceptable for survival, with minimum reproductive costs i.e. reducing the negative impact of the infection on host fitness [99]. This is the case in the trypanosome-infected salivary gland where the parasites number is controlled at a level that has no impact on the fly survival during the experimental period of more than 40 days.

As mentioned above, the differential expression analysis indicates the trypanosome infection causes a serious tissue damage in the salivary glands. This cell damage can elicit danger signals (like *pvf1* in our data set) that subsequently cause a triggering of an immune response in order to keep the infection under control. These damage signals are generated during host-pathogen tissue interactions either by mechanical or proteolytic damage. An insect immune response in the infected tissue can thus be activated by recognition of both non-self and molecular by-products of tissue damage [97, 98]. The upregulation of genes involved in the cytoskeleton dynamics (formation of cytoskeleton filaments), cell repair and septic junction formations in the trypanosome-infected glands suggest that the parasite tight attachment and the dense parasite packing in the gland has a severe impact on the epithelial structure integrity. A similar strong epithelial response was reported in the mosquito midgut epithelium when invaded by the *Plasmodium* ookinetes [75, 100]. In contrast, *Plasmodium* sporozoite invasion of the salivary glands did not have a drastic impact on the epithelial cells [101]. The *T. brucei* attachment to the salivary gland epithelium is an essential event for the parasite in the maintenance of its life cycle as it ensures that the tsetse saliva remains trypanosome-infected during the whole life span of the fly. The tight attachment of the parasite flagellum to the gland epithelial cells is described as a hemi-desmosome-like junctional complex [96] mediated by an unidentified ligand-receptor interaction. From our data, it appears that this junctional complex has a severe impact on the salivary gland epithelium integrity.

The strong upregulation of a broad set of genes involved in detoxification can be explained by the need to prevent the gland environment to become too toxic for the epithelial and secretory cells. Indeed, the gland is densely packed with active, metabolizing and also dying parasites that can be assumed to drastically change the biochemical characteristics of the glands such as lower pH and increase of ROS. The fact that the normal functioning of the infected salivary gland is significantly hampered indicates that this detoxification is only partially successful leaving the cells to function in suboptimal physiological conditions. A cascade of alterations of detoxifying gene expression was also observed in the *Plasmodium-Anopheles* system where invasion of the midgut epithelium and the hemocoel resulted in the modulation of detoxifying genes in midgut and fat body of the mosquito [102].

## Conclusions

This study confirmed that the *T. brucei* population in the tsetse salivary gland has a negative impact on its functioning and on the integrity of the gland epithelium. Our detailed and robust RNA-seq data analysis indicates the induction of a strong local tissue response allowing the fly to tolerate the trypanosome infection in the glands. This tolerance implies the control of i) the epithelial cell damage, ii) the ROS intoxication of the cellular environment and iii) the parasite infection. The upregulated expression of some gene categories in the salivary glands by a trypanosome midgut infection suggests a possible anticipatory response of the salivary gland environment before the parasites reach this tissue. These findings contribute to a better understanding of the biological impact of the sleeping sickness parasite on the tsetse fly and how this insect vector keeps this impact under control.

## Additional files

Additional file 1: Table S1. List of genes added to the GmorY1 assembly. (XLS 27 kb)
Additional file 2: Table S2. Real-time quantitative RT-PCR validation of RNA-seq data. (XLS 39 kb)
Additional file 3: Table S3. List of differentially expressed transcripts between SG + MG+ vs SG-MG-. (XLS 2344 kb)
Additional file 4: Table S4. List of differentially expressed transcripts between SG + MG+ vs SG-MG+. (XLS 1305 kb)
Additional file 5: Figure S1. Heat map showing the differentially expressed transcripts shared between experimental conditions. The heat maps were obtained by plotting the mean of normalized read counts (scaled by row and hierarchical clustered) in the three infection conditions. Colors display z-scores from −2 (low expression: dark blue) to 2 (high expression: red) for normalized gene expression values. (PDF 107 kb)
Additional file 6: Table S5. List of differentially expressed transcripts shared between experimental conditions. (XLS 838 kb)
Additional file 7: Figure S3. The Immune Deficiency (Imd) signaling pathway in tsetse fly. The yellow squares represent members of the pathway in *Glossina morsitans* genome; the triangle inducible negative regulator; the hexagon constitutive negative regulator; the transparent figures indicate a transcript that had no reads or the orthologue was not annotated in the tsetse fly genome. A broken line designates not a clear interaction. ROS: reactive oxygen species; DAP-PGN: diaminopimelic acid peptidoglycan; P-phosphorylation; Ub- ubiquitination. The part of the figure with *pirk* expression was adapted after [103]. (PDF 220 kb)
Additional file 8: Figure S4. The JAK/STAT signaling pathway in tsetse fly. The yellow squares represent members of the pathway in *Glossina morsitans* genome; the hexagon constitutive negative regulator; the transparent figures indicate a transcript that had no reads or the orthologue was not annotated in the tsetse fly genome. A broken line designates not a clear interaction. (PDF 74 kb)
Additional file 9: Figure S5. The Toll signaling pathway in tsetse fly. The yellow squares represent members of the pathway in *Glossina morsitans* genome; the hexagon constitutive negative regulator; the transparent figures indicate a transcript that had no reads or the orthologue was not annotated in the tsetse fly genome. (PDF 71 kb)
Additional file 10: Figure S2. Heat map showing the affected salivary gland transcripts in midgut only *T. brucei-*infected flies*.* The heat maps were obtained by plotting the mean of normalized read counts (scaled by row and hierarchical clustered) in the three infection conditions. Colors display z-scores from −2 (low expression: dark blue) to 2 (high expression: red) for normalized gene expression values. (PDF 190 kb)
Additional file 11: Table S6. List of transcripts that showed increased expression in SG-MG+ due to the presence of the parasite in the midgut. (XLS 306 kb)

## Abbreviations

AMP: Antimicrobial peptide
CTL: C-type lectin
CYP: P450-cytochrome
ECM: Extracellular matrix
GO: Gene Ontology
GTS: Glutathione S transferase
MG: Midgut
NOS: Nitric oxide synthase
﻿PCR: Polymerase chain reaction
﻿PRR: Pattern recognition receptor
RNS: Reactive nitrogen species
ROS: Reactive oxygen species
RT: Reverse Transcription﻿
SG: Salivary gland
SJ: Septate junction
SP: Serine protease
SPH: Serine proteases homologue
TEP: Thioester-containing protein
