# Supplementary figures and images for: Tsetse fly tolerance to T. brucei infection: transcriptome analysis of trypanosome-associated changes in the tsetse fly salivary gland
Source: BMC Genomics. 2016 Nov 25;17:971. doi: 10.1186/s12864-016-3283-0 (PMC5123318; doi:10.1186/s12864-016-3283-0)

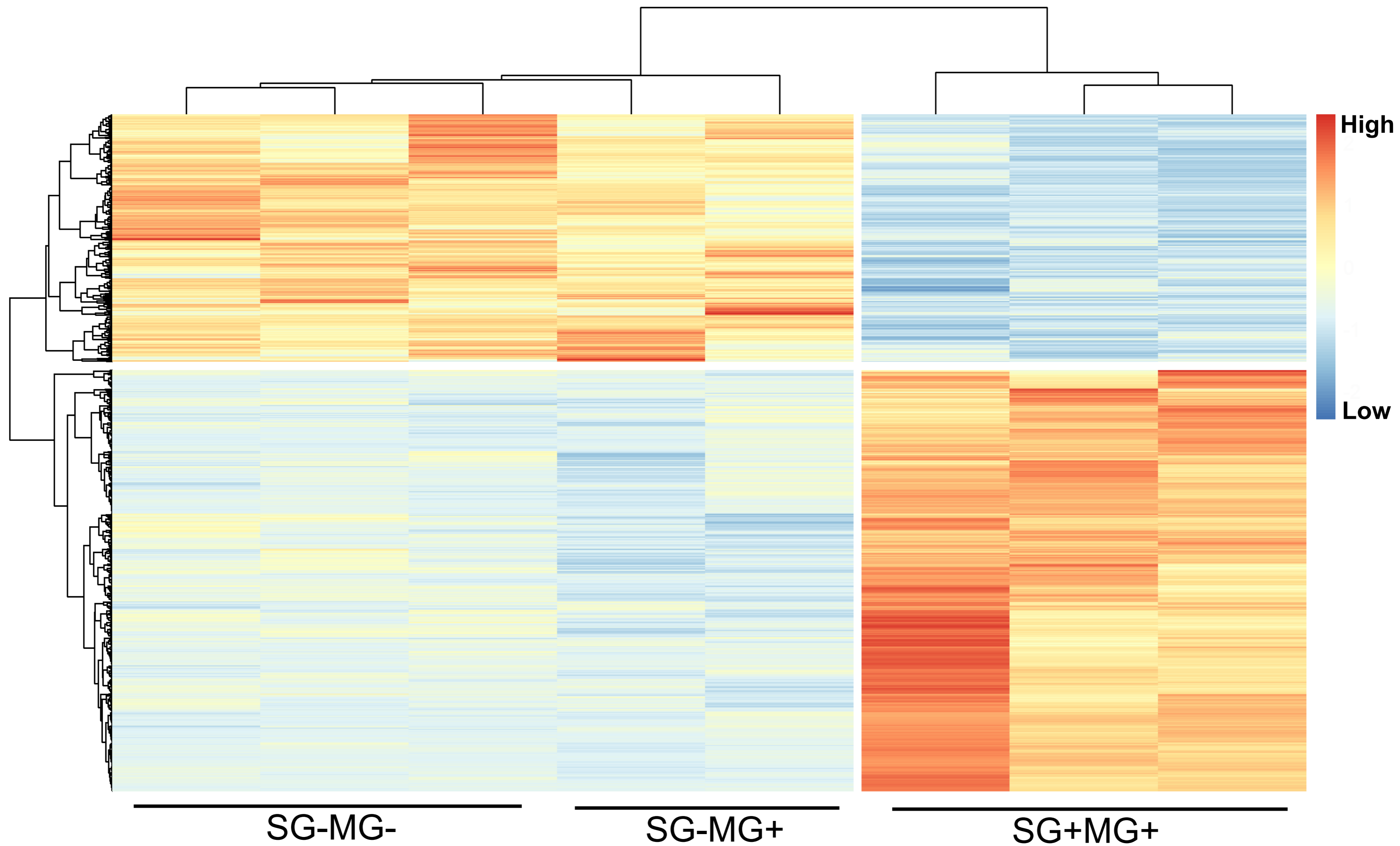

Supplement: Additional file 5: Figure S1. — Heat map showing the differentially expressed transcripts shared between experimental conditions. The heat maps were obtained by plotting the mean of normalized read counts (scaled by row and hierarchical clustered) in the three infection conditions. Colors display z-scores from −2 (low expression: dark blue) to 2 (high expression: red) for normalized gene expression values. (PDF 107 kb) [file 12864_2016_3283_MOESM5_ESM.pdf]

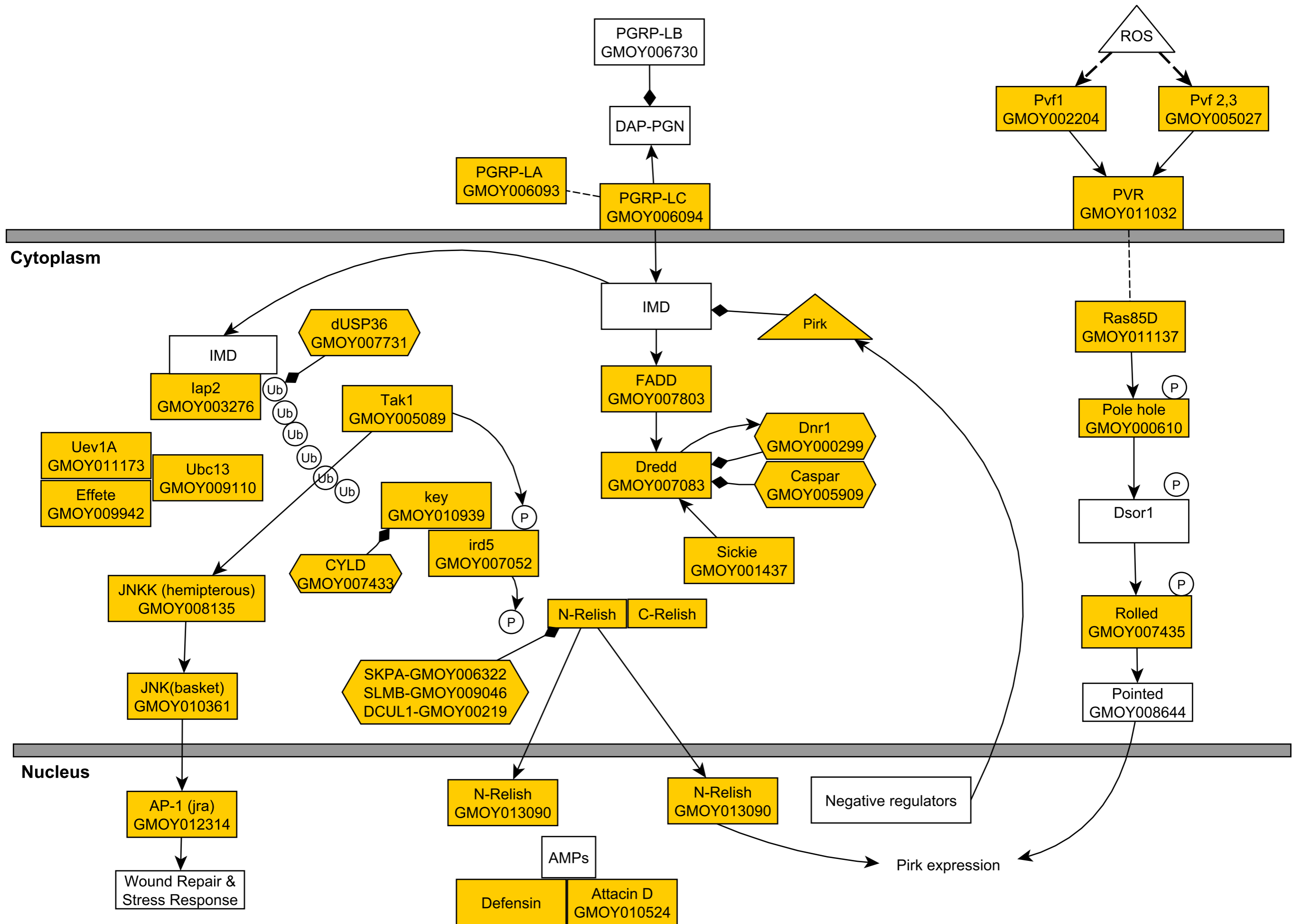

Supplement: Additional file 7: Figure S3. — The Immune Deficiency (Imd) signaling pathway in tsetse fly. The yellow squares represent members of the pathway in Glossina morsitans genome; the triangle inducible negative regulator; the hexagon constitutive negative regulator; the transparent figures indicate a transcript that had no reads or the orthologue was not annotated in the tsetse fly genome. A broken line designates not a clear interaction. ROS: reactive oxygen species; DAP-PGN: diaminopimelic acid peptidoglycan; P-phosphorylation; Ub- ubiquitination. The part of the figure with pirk expression was adapted after [103]. (PDF 220 kb) [file 12864_2016_3283_MOESM7_ESM.pdf]

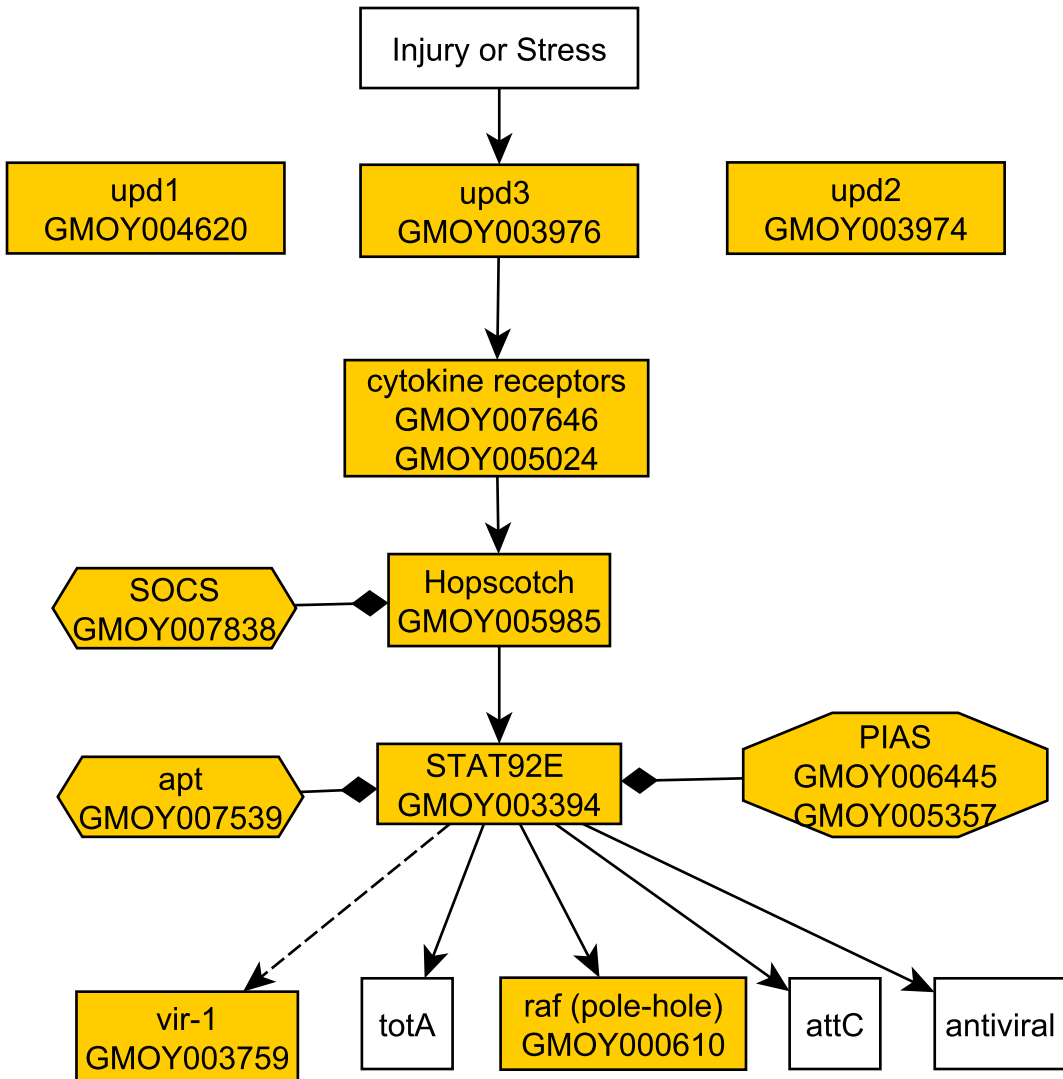

Supplement: Additional file 8: Figure S4. — The JAK/STAT signaling pathway in tsetse fly. The yellow squares represent members of the pathway in Glossina morsitans genome; the hexagon constitutive negative regulator; the transparent figures indicate a transcript that had no reads or the orthologue was not annotated in the tsetse fly genome. A broken line designates not a clear interaction. (PDF 74 kb) [file 12864_2016_3283_MOESM8_ESM.pdf]

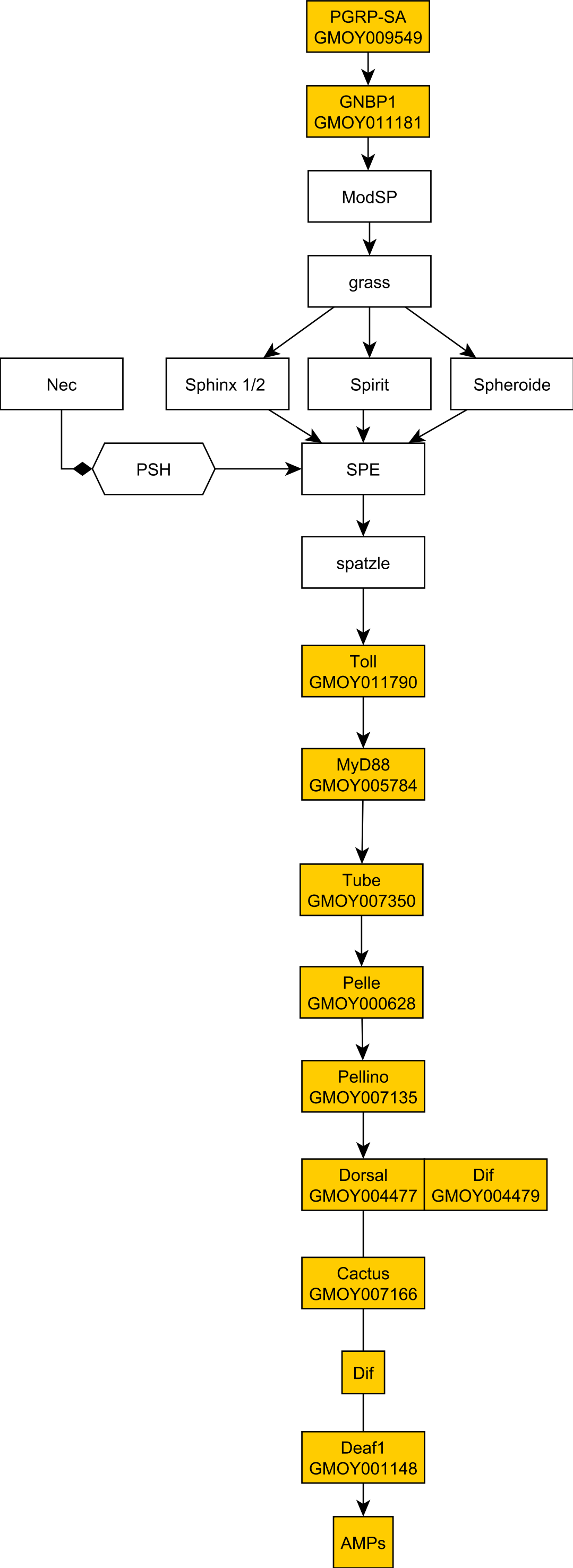

Supplement: Additional file 9: Figure S5. — The Toll signaling pathway in tsetse fly. The yellow squares represent members of the pathway in Glossina morsitans genome; the hexagon constitutive negative regulator; the transparent figures indicate a transcript that had no reads or the orthologue was not annotated in the tsetse fly genome. (PDF 71 kb) [file 12864_2016_3283_MOESM9_ESM.pdf]

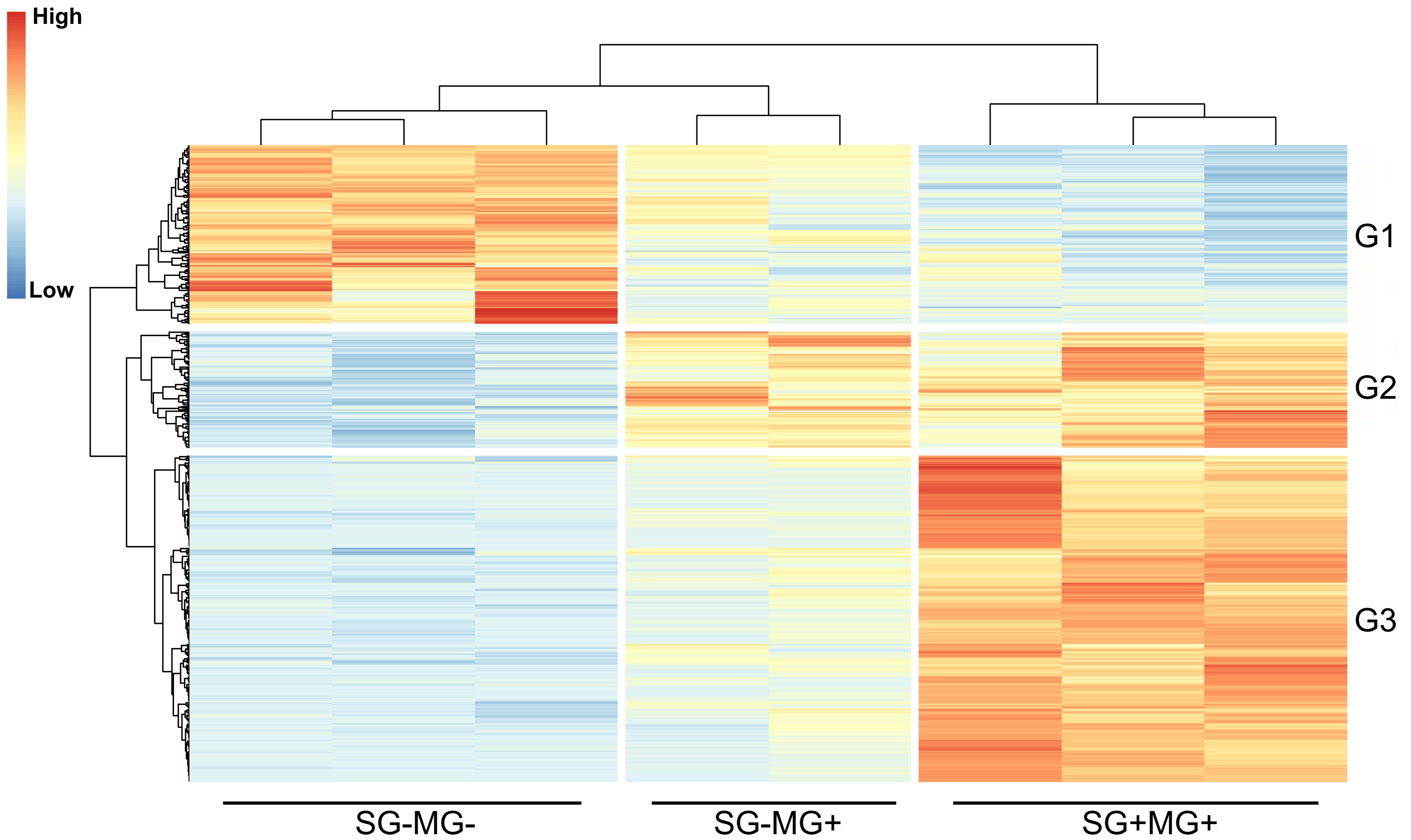

Supplement: Additional file 10: Figure S2. — Heat map showing the affected salivary gland transcripts in midgut only T. brucei-infected flies. The heat maps were obtained by plotting the mean of normalized read counts (scaled by row and hierarchical clustered) in the three infection conditions. Colors display z-scores from −2 (low expression: dark blue) to 2 (high expression: red) for normalized gene expression values. (PDF 190 kb) [file 12864_2016_3283_MOESM10_ESM.pdf]
